# Proton Arc Therapy vs Interstitial HDR Brachytherapy in Gynecologic Cancer with Parametrial/pelvic Side Wall Extension

**DOI:** 10.14338/IJPT-22-00013.1

**Published:** 2022-06-28

**Authors:** ByongYong Yi, Sina Mossahebi, Arezoo Modiri, Elizabeth M. Nichols, Mariana Guerrero, Narottam Lamichhane, Pranshu Mohindra

**Affiliations:** 1 Department of Radiation Oncology, University of Maryland School of Medicine, MD, USA; 2 Proton Treatment Center, Baltimore, MD, USA

**Keywords:** interstitial brachytherapy, proton arc therapy, locally advanced gynecologic cancer

## Abstract

**Purpose:**

To investigate whether volumetric-modulated proton arc therapy (VPAT) plans generate comparable doses to organs at risk (OARs) compared with interstitial high–dose-rate (iHDR) brachytherapy for patients with gynecologic cancer with disease extension to parametrial/pelvic side wall, who are not eligible for the aggressive procedure.

**Materials and Methods:**

VPAT delivers proton arc beams by modulated energies at the beam nozzle while maintaining the same incident energy to the gantry during the arc rotation. Plans of 10 patients previously treated with iHDR brachytherapy for high-risk clinical treatment volumes (HRCTV; 31.8–110.6 cm^3^; lateral dimensions, 4.2–5.6 cm) were selected and compared with VPAT plans. VPAT plans for each patient were designed using a 152- to 245-MeV range of energy-modulated proton beams.

**Results:**

HRCTV coverage of the VPAT plans was comparable to that of the iHDR plans, with V150% showing no statistical differences. On average, the V100% and V90% of VPAT plans were higher than those of the iHDR plans, 95.0% vs 91.9% (*P* = .02) and 98.6% vs 97.5% (*P* = .02), respectively. D100 was also 17% higher for the VPAT plans (*P* = .03). On average, the D_2cm^3^_ of bladder, rectum, and small bowels in the VPAT plans were considerably lower than those in iHDR plans (by 17.4%, 35.2%, and 65.6%, respectively; *P <* .05 for all OARs).

**Conclusion:**

VPAT–generated plans were dosimetrically superior to those with HDR brachytherapy with interstitial needles for locally advanced gynecologic cancer with parametrial/pelvic side wall disease extension. Dosimetrically, VPAT provides a noninvasive alternative to iHDR brachytherapy with a superior dosimetric profile.

## Introduction

Standard treatment for locally advanced cervical cancer is concurrent chemoradiotherapy, and radiotherapy consists of external-beam radiotherapy to the pelvis and brachytherapy. Brachytherapy is an essential component of definitive radiotherapy because it can deliver very high dose to the cervical tumor with minimizing doses to the organs at risk (OARs). In multiple, large, national, retrospective data sets in the US, the use of brachytherapy declined between 2003 and 2011, whereas the use of intensity-modulated radiotherapy or stereotactic body radiotherapy instead increased during this period. These data suggested that omission of brachytherapy had a strong negative impact on survival. Therefore, several guidelines for cervical cancer recommend neither stereotactic body radiotherapy nor intensity-modulated radiotherapy are a suitable substitute for brachytherapy and should only be considered for those determined to be ineligible because of complex medical factors [1–3]. Recent developments in image-guided adaptive brachytherapy have improved treatment results in locally advanced cervical cancer [4]. The conventional tandem-and-ovoid or -ring approach, however, is often inadequate for treating large or irregular-shaped tumors. Interstitial high–dose-rate (iHDR) brachytherapy, a technique that combines intracavitary applicators and interstitial needles, is increasingly used [5, 6]. Despite clinical advantages, iHDR brachytherapy has multiple constraints and challenges in daily clinical practice. Brachytherapy is an invasive procedure, especially when using interstitial needles, which typically requires procedures to be done in the surgical settings under anesthesia. Plan quality is highly operator and setup dependent [7].

**Figure 1.**
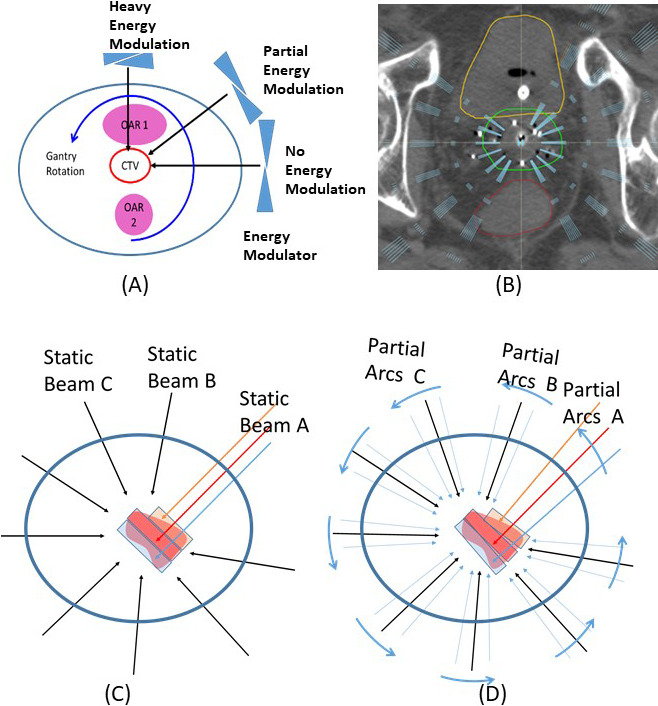
Principle of Volumetric–modulated proton arc therapy (VPAT). (A) Energy is modulated while gantry is moving, (B) distribution of beams in patient 1, (C) static plan optimization of 9 to 11 proton beams, and (D) each energy layer is assigned as a single beam of 1° interval to make an arc plan.

Volumetric-modulated arc therapy (VMAT) has been suggested and implemented as a treatment alternative to gynecologic brachytherapy [8–11]. Relevant studies have used cylinders [8, 9] and tandem-and-ring or -ovoid [10, 11] configurations. These studies have shown target coverage with VMAT plans is comparable to that of HDR brachytherapy in terms of coverage but with more uniform dose homogeneity across the target volume. OAR doses were also reported to be comparable. These studies concluded that VMAT can be a noninvasive alternative to standard intracavitary brachytherapy. However, the lack of high-dose heterogeneity that is otherwise seen in brachytherapy plans is likely one of the key limitations supporting routine use of VMAT. Further, these studies did not evaluate feasibility of using VMAT in patients with bulky parametrial/side wall extension. Many studies have demonstrated that proton treatment offers approximately 50% to 60% less integral dose than comparable photon treatment [12–14]. Learning from the photon VMAT comparison studies and from the fact that proton offers a less-integral dose, we expect that proton arc therapy might be superior to photon arc as an alternative to iHDR brachytherapy. Several practical and achievable proton arc techniques have been suggested recently [15–18]. Langner et al [16] and Modiri et al [17] have proposed a proton arc therapy concept using an external energy modulator (EEM). Langner et al [16] suggested to deliver to a plane at each different gantry angle, while Modiri et al [17] suggested volumetric dose at each sector of gantry angles, which is called volumetric-modulated proton arc therapy (VPAT; **Figure 1A**). The concept is briefly summarized here. A key challenge in proton therapy and even more so in proton arc therapy is switching energies during beam delivery because the skin-to-target depth changes continuously with gantry movement [19]. VPAT employs continuous energy-modulated proton beams using an EEM, as shown in **Figure 1**. Thickness of the modulator in the EEM at any gantry angle is determined by calculating the depth of the treatment plane from the surface of the gantry angle of the treatment delivery moment. The energy-modulated proton beam reaches the determined position of each gantry angle while incident proton energy is maintained at the same level from the beginning to the end of the arc treatment delivery. Monitor units are programmed in synchrony with the gantry angles and the delivering proton energy, as does the thickness of the EEM. To perform intensity-modulated proton therapy in VPAT, the optimal energy is determined at each gantry angle and spot intensities are optimized on the treatment planes of the determined energies.

In addition to the potential for improved target coverage, proton arc therapy may also offer controlled heterogeneity in the target mimicking the high-dose distributions of intracavitary brachytherapy. In this study, we explored whether the VPAT can generate clinically acceptable plans that are comparable to iHDR plans for gynecologic cancer with parametrial/pelvic side wall extension for patients who are ineligible for the iHDR procedure due to complex medical factors. Further, we evaluate if VPAT can help reduce dose to OARs, such as the bladder, rectum, small bowel, and sigmoid colon, which might have a bearing on reducing the risk of toxicity. This would have the potential to lead to a decreased risk of fistula formation, a known but problematic side effect for patients.

## Materials and Methods

### Patient Data

In our institutional review board–approved study, we analyzed 10 iHDR gynecologic brachytherapy plans, as summarized in **Table 1**. The median dimensions for high-risk clinical target volumes (HRCTVs) were 5.0 (maximum [max], 5.6; minimum [min]: 4.4), 4.2 (5.2, 3.2), and 8.4 (11.2, 5.6) cm for the X, Y, and Z directions, respectively. The median HRCTV was 57.3 cm^3^ (max, 110.6; min, 31.8). An intrauterine applicator and 6 to 18 needles with an interstitial template were used. Total doses of 20 to 28 Gy in 4 to 5 fractions were prescribed to the HRCTV. Oncentra V3 (Elekta, Stockholm, Sweden) planning system was used to generate clinical plans.

**Table 1.** Interstitial high-dose-rate brachytherapy characteristics for study cases.

**Number**	**Anatomy**	**Applicator**	**HRCTV, cm^3^**	**HRCTV size, cm**	**Needles, n**	**External dose, cGy**	**HDR dose, cGy/Fx**
1	EndoCervix	Syed	75.7	4.9 × 3.7 × 11.2	15	6660	2000/5
2	Cx	Syed	110.6	5.6 × 4.5 × 9.1	18	4500 + 1000	2625/5
3	Cx	Syed	81.7	5.1 × 3.4 × 9.5	16	4500 + 1000	2250/5
4	Vagina	Syed	79.0	5.0 × 4.6 × 7.0	16	5040 + 540	2250/5
5	Endometrium	Syed	54.8	5.3 × 3.2 × 8.6	10	No ext	2500/5
6	Vagina	Syed	87.6	5 × 3.8 × 8.7	14	5040 + 840	2500/5
7	EndoCervix	Venezia	64.1	4.9 × 5.2 × 5.6	7	4500 + 900	2800/4
8	Cx	Venezia	32.0	4.4 × 4.7 × 8.2	7	4500	2800/4
9	Cx	Venezia	50.5	5.6 × 4.6 × 6.8	6	4500 + 1000	2800/4
10	Cx	Syed	47.0	4.7 × 3.7 × 5.7	10	4500 + 540	2250/5

**Abbreviations:** HRCTV, high-risk clinical treatment volumes; HDR, high-dose rate.

### VPAT Planning for iHDR Cases

The RayStation V8.0A (RaySearch, Stockholm, Sweden) planning system was used to generate VPAT plans. A script and workflow steps were developed to simulate the VPAT optimization plans and are summarized here as follows:

Step 1: Generate a plan with static multiple beams with different gantry angles (**Figure 1C**).
  1. Nine to 11 proton beams, evenly spaced at approximately 30° intervals, were generated. No beams were assigned to anterior and posterior directions to avoid beams directed to the bladder or rectum. Ranges of gantry angles of avoidance were manually selected.
  2. Single-field optimization was used, if possible.
  3. Planning objectives were to achieve 95% of HRCTV receives 100% of the prescribed dose and dose to 2 cm^3^ of bladder, rectum, and small bowel limited to or less than iHDR doses.
Step 2: Convert the optimized plan to an arc plan (**Figures 1B** and **1D**)
  1. Each energy layer was split into 1° intervals to create an arc plan.
  2. Dose distributions were recalculated to determine whether the plan maintained the same dose distribution as the static-field plan,
  3. A robustness test of 3-mm/3.5% was applied.

Constraints for heterogeneous dose distributions in the target were applied to achieve a V150% in the HRCTV like that in the HDR plans.

### Statistical Analysis

All the VPAT plans were normalized to deliver 100% of the prescribed dose to 95% of HRCTV. The plans between the VPAT and the iHDR were compared. HRCTV dose parameters such as V150, V100, V90, and D100 were used to compare the plans and D_2cm^3^_ of bladder, rectum, and small bowels were used for OAR dose comparisons. Student *t* test was used to determine the significance between the 2 plans using a 95% confidence level.

## Results

Energies used in the planning were in the range of 152 to 220 MeV, with a maximum variation of energy modulation of 55 MeV per patient, except for a single, large patient in whom energy ranged between 175 and 245 MeV, with maximum energy variation of 70 MeV.

**Figure 2.**
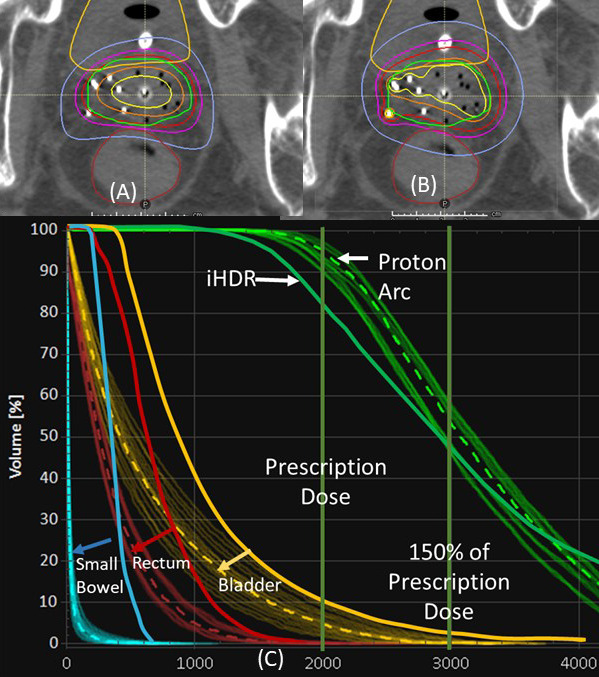
Plan comparison of (A) volumetric-modulated proton arc therapy (VPAT) and (B) interstitial high-dose-rate (iHDR) plans of patient 1 and (C) dose–volume histograms (DVH) comparisons. Solid thick lines represent the DVH curves from iHDR plan and dotted are those from VPAT plan. Thin lines are from 3mm/3.5% robust plans of VPAT. VPAT achieved lower organ-at-risk doses with better high-risk clinical target volume coverage in the DVH. In the VPAT robust plans, V100 of the worst-case scenario decreased by 5%, which was still comparable to the iHDR plan.

**Figure 2** shows typical dose distributions and dose–volume histograms (DVHs) of the iHDR and VPAT plans. Heterogeneous dose distributions in the HRCTV, V150 were similarly achieved (average difference less than 8%) with the VPAT and iHDR plans. **Table 2** summarizes the dosimetric results for targets and OARs. No significant differences in the V150% were observed between the 2 plans. In contrast, significant differences were seen in D100, D90, V100, and V90 between the 2 plans. V100% was always maintained at 95% for VPAT plans, whereas these percentages varied from 82.9% to 96% for iHDR plans. V90% and D100 for the VPAT plans were higher than those for iHDR plans. The differences for V100% and V90% were statistically significant but the differences were only 2% to 3%. The worst scenario in the robustness tests with 3-mm/3.5% for VPAT reduced V100% by 5% on average. This was still comparable to the performance of iHDR.

**Figure 3.**
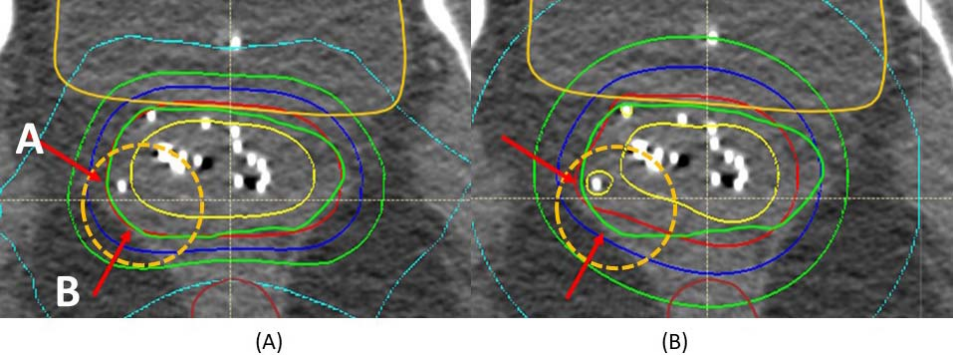
Volumetric-modulated proton arc therapy (VPAT) (A) and interstitial high-dose-rate (iHDR) (B) plans in patient 6. Because the source was located near arrow A, the target was well covered in both plans. The iHDR plan showed less coverage near arrow B, whereas the VPAT plan covered the target volume well.

**Table 2.** Plan comparisons between high-risk clinical target volume (HRCTV) coverage and organs at risk (OARs) doses

**Number**	**HRCTV**								**OARs**					
	**V150 (%)**		**V100 (%)**		**V90 (%)**		**D100 (cGy)**		**Bladder D_2cm^3^_**		**Rectum D_2cm^3^_**		**Sm Bowel D_2cm^3^_**	
	**HDR**	**Proton**	**HDR**	**Proton**	**HDR**	**Proton**	**HDR**	**Proton**	**HDR**	**Proton**	**HDR**	**Proton**	**HDR**	**Proton**
1	48.1	49.0	82.9	95.0	90.1	98.0	916	1183	2416	1978	1381	1114	453	62
2	47.9	63.0	93.6	95.0	97.5	97.9	1630	1784	2360	1838	1825	1601	815	89
3	49.5	68.5	90.2	95.0	96.0	98.0	1481	1883	2267	1769	1479	1003	697	71
4	31.6	36.0	91.4	95.0	96.9	100.0	1391	2095	1407	1443	1269	1043	<10	<10
5	91.6	34.0	92.7	95.0	95.0	99.1	1183	1858	2279	1193	1087	840	472	55
6	60.0	65.0	93.7	95.0	97.1	99.5	1508	2386	2338	2209	1488	620	734	248
7	53.0	48.0	88.0	95.0	95.0	97.7	1929	2295	1861	1324	1359	567	<10	<10
8	60.8	49.5	94.3	95.0	98.9	98.6	2268	1916	2026	1499	1282	744	1145	430
9	57.9	46.0	96.0	95.0	99.1	98.5	2086	1873	2112	1990	1676	730	620	63
10	63.5	63.7	96.0	95.0	99.7	98.9	1688	1643	<10	<10	1410	947	389	62
Mean	56.4	52.3	91.9	95.0	96.5	98.6	1608	1892	2118	1694	1426	921	666	135
*P* value	.28		.02		.02		.03		.002		<.001		<.001	

**Abbreviation:** HDR, high-dose rate.

All OARs received significantly lower doses (*P* < .05 for all) in VPAT than in iHDR plans. D_2cm^3^_ values were reduced by 17.4%, 35.2%, and 65.6% for bladder, rectum, and small bowels, respectively. Small bowel or bladder doses of some cases in Table 2 were less than 10 cGy for both plans, and these cases were not used in calculating *P* values.

## Discussion

In HDR brachytherapy, high doses are delivered to tumor while limiting doses to OARs (especially those proximate to the target) by generating a steep dose gradient. However, HDR brachytherapy is an invasive procedure, with interstitial HDR procedures being even more invasive. Interstitial HDR is associated with increased health care costs related to an inpatient hospitalization. Moreover, dose optimization is not feasible for iHDR planning in areas with no needles—where there are no needles, there is no doses. In the case shown in **Figure 2B**, for example, it is not possible for an iHDR plan to cover the HRCTV unless a needle is at the region of interest whereas VPAT plans do not have that limitation for adjusting the dose distribution, as shown in **Figure 2A**. Adding needles during the planning procedure is not a feasible option; the depth of the needles can be adjusted only at the imaging phase [20]. During planning, the dwell times of the source positions in the needles can be flexibly adjusted. Allocating needles to the right places is always a challenging task. For the 10 cases used in this study, 119 needles were inserted, and 20 needles were not used during planning (i.e., on average, 12 needles per patient were used and 2 needles were not used). Analysis of HRCTV volume vs number of needles indicates that 1.7 needles are used in the planning for every 10 cm^3^ of treatment volume and shows a strong correlation (0.74) between treatment volume and number of needles used in planning. Having more needles is always better than fewer in planning, increasing flexibility in optimization. Not all inserted needles were used in planning. When including both used and unused needles the total becomes 2.0 needles/10 cm^3^. A fast dose gradient is expected in photon VMAT when the target size is small. For typical brachytherapy cases with cylinders, the diameter of the target is usually less than 4 cm. Planning comparison studies between VMAT and HDR typically use cylinders of 2 to 4 cm in diameter to generate comparable dose gradients outside the target [8–11]. In iHDR brachytherapy, however, the diameter of the HRCTV is 5 cm or larger (**Table 1**). As it is shown for the cases of cylinders, rings, or ovoids, VMAT might have the advantage of flexibility in tailoring dose distributions in the target when treating gynecologic cancer with parametrial or pelvic side wall extension, because it does not have the limitation of needle locations. However, because of the size of the target, a slow dose gradient outside the target volume is expected. Proton beam treatment promises less integral dose than photons [12–14] because the proton beam exit component is negligible. Therefore, proton arc therapy has a dosimetric advantage in pursuing alternative to iHDR brachytherapy than photon VMAT. Average dose of 10 cm from the center showed less than 5% of the prescribed dose. Clinical meaning of this low dose needs to be investigated in the further study. For iHDR plans, the dose calculation range is often less that 10 cm from the center, which means that the iHDR dose far from the center of the HRCTV is regarded as clinically insignificant.

It was the goal of this study to determine whether a proton arc plan can achieve dose quality equivalent to that of an iHDR plan. An exact comparison between an external-beam plan and an HDR plan is challenging because of the following factors: (1) dose heterogeneity; (2) patient size dependency of OAR doses; (3) target size dependency of dose gradients; and (4) robustness evaluation. Dose is heterogeneous across the target in a typical HDR plan. A high dose in the target area may increase the chance of tumor eradication. To achieve this effect, the VPAT plans were designed to have a higher dose at the center of the target. A limitation of this paper is still less dose heterogeneity across the HRCTV than that of iHDR. For a HDR plan, the dose at the source dwell points can easily be several times higher than the prescribed dose. It was not the goal of proton arc planning to achieve extremely high doses in the target. For practical reasons, VPAT plans were designed to generate V150 like HDR plans. As we can see in **Table 2**, dose heterogeneity in the HRCTV in the VPAT plans is comparable to that of iHDR plans. Further study may be needed to determine how high the proton plans should go to achieve the same clinical effect as iHDR brachytherapy; this is beyond the scope of this paper. Patient size is an important contributing factor to integral dose for external-beam treatment, whereas the size is not an issue for HDR brachytherapy.

Another limitation of this paper is that intrafractional organ motion has not been considered in 3-mm robust test. Haripotepornkul et al [21] reported that the intrafractional motion range is average 2.9 mm in anterior-posterior direction and up to 15 mm for each direction during intensity modulated radiation therapy (IMRT). Holding organ motions during the treatments is one of the key tasks to make the proton arc therapy as an alternate solution. Either applying fiducial marker object in the uterus [22], or vaginal fixation device [23] can be considered for this purpose. Further study on developing the method and its dosimetric effect is necessary.

Dosimetric effects of interfractional displacement of source or needle positions is often ignored in iHDR treatment. Tambas et al [24] reported that 68% of needles shifted 2 ± 2.3 mm, and other researchers have reported shifts up to 5 mm [25–27]. Such shifts alter dose distribution and are comparable to the typical robustness range of a proton plan of 3 mm/3.5%. Degradation of V100 up to 5% for a worst-case scenario of VPAT has been identified in this study. It is still comparable to iHDR plans, with the assumption of no displacement of source positions during the iHDR treatments. It is not standard practice to routinely evaluate plan robustness in iHDR. Plan quality will be decreased if needle position uncertainty is included in iHDR plan evaluation.

**Figure 4.**
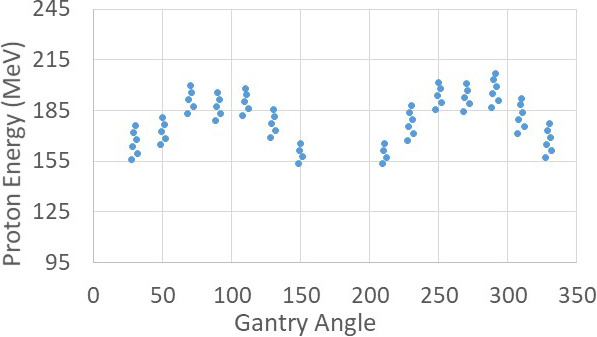
Energy distributions per gantry angles for the high-efficiency volumetric-modulated proton arc therapy plan in Case 10. Energy is modulated for every gantry angle.

Two other types of proton arc delivery methods have been introduced in literature. Bertolet and Carabe [18] described proton monoenergetic arc therapy (PMAT). PMAT delivers the same energy for a segment of angles and then switches the energy. Ding et al [15] proposed spot-scanning proton arc (SPArc) therapy, which optimizes control points and energy layers by iterations. Both methods require frequent energy switching. SPArc tries the energy-switching downward to reduce delivery time, since increasing energy takes longer than decreasing. As seen in **Figure 4**, frequent energy up and down is required to deliver plans suggested in this study, making both PMAT and SPArc extremely inefficient or longer treatment time due to frequent energy switching. VPAT, however, provides a flexible solution of energy change, because it uses a single energy throughout the arc and delivered energy is modulated by an EEM for which energy switching time is negligible, regardless of increasing or decreasing energies. Among the proposed methods, VPAT is the most efficient for delivering proton arc plans as an alternative to HDR brachytherapy, considering energy switching efficiency.

## Conclusion

Proton arc treatment plans for patients with locally advanced gynecologic cancer with parametrial or pelvic side wall extension showed equivalent and acceptable dosimetric results compared with iHDR plans. HRCTV coverage was comparable or better with the proton plans for V150%, V100%, V90%, and D100%. All OAR doses, including those to rectum, bladder, and small bowels, showed significant reductions (*P* < .05 for all OARs). VPAT can provide a noninvasive alternative to iHDR brachytherapy with equivalent dosimetric profile.
